# Build-Up of a 3D Organogel Network within the Bilayer Shell of Nanoliposomes. A Novel Delivery System for Vitamin D_3_: Preparation, Characterization, and Physicochemical Stability

**DOI:** 10.1021/acs.jafc.0c06680

**Published:** 2021-02-22

**Authors:** Fatemeh Ghiasi, Mohammad Hadi Eskandari, Mohammad-Taghi Golmakani, Ramón G. Rubio, Francisco Ortega

**Affiliations:** † Department of Food Science and Technology, School of Agriculture, Shiraz University, Shiraz 71946-84636, Iran; ‡ Departamento de Química Física, Facultad de Ciencias Químicas, Universidad Complutense de Madrid, Ciudad Universitaria S/n, Madrid 28040, Spain; § Instituto Pluridisciplinar, Universidad Complutense de Madrid, Paseo Juan XXIII 1, Madrid 28040, Spain

**Keywords:** nanoliposome, organogel, vitamin D_3_, encapsulation, EPR, DLS, zeta potential

## Abstract

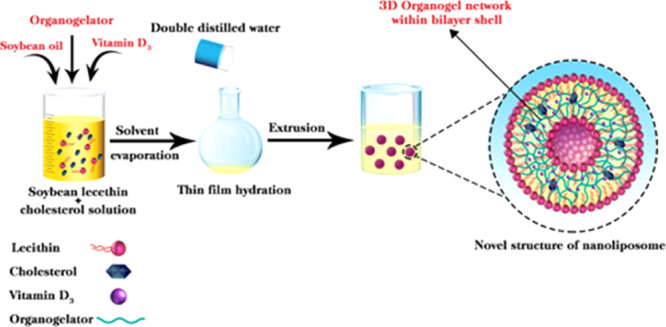

The inherent thermodynamic instability of liposomes during production and storage has limited their widespread applications. Therefore, a novel structure of food-grade nanoliposomes stabilized by a 3D organogel network within the bilayer shell was developed through the extrusion process and successfully applied to encapsulate vitamin D_3_. A huge flocculation and a significant reduction of zeta potential (−17 mV) were observed in control nanoliposomes (without the organogel shell) after 2 months of storage at 4 °C, while the sample with a gelled bilayer showed excellent stability with a particle diameter of 105 nm and a high negative zeta potential (−63.4 mV), even after 3 months. The development of spherical vesicles was confirmed by TEM. Interestingly, the gelled bilayer shell led to improved stability against osmotically active divalent salt ions. Electron paramagnetic resonance confirmed the higher rigidity of the shell bilayer upon gelation. The novel liposome offered a dramatic increase in encapsulation efficiency and loading of vitamin D_3_ compared to those of control.

## Introduction

Liposomes are vesicular structures composed of phospholipids that self-assemble into one or more concentric bilayers by dispersing in an aqueous medium.^1^ Among the lipid-based delivery systems, liposomes are considered as the most efficient carriers in the formulation of pharmaceutical and cosmetic products to encapsulate unstable active multi-components, to enhance the oral bioavailability of poorly water-soluble compounds, to provide a controlled release, and to extend the circulation lifetime of compounds.^2,3^ This fact can be attributed to their structural flexibility, particle size, chemical composition, and fluidity or permeability of the lipid bilayer versatility. Moreover, liposomes are biodegradable and biocompatible due to the structure and physicochemical similarity to the cell membrane phospholipids, causing no harmful effects on the human health.^4^ However, the application of liposomes in the food industry is still limited by their poor stability over a long storage period due to degradation, fusion, aggregation, or sedimentation and high tendency to lose entrapped compounds during storage as a function of osmotic pressure in the presence of food components or additives, such as sugars or salts.^5−7^ The physical stability of liposomes strongly depends on molecular ordering, packing, and dynamics of acyl chains in the bilayer, charge intensity, as well as the physical state–gel or fluid–and composition of lipids.^8−10^ Therefore, many efforts have been made to overcome these challenging tasks, including liposome coating with hydrogel networks,^11^ surface modification of vesicles with polymeric matrices using an electrostatic layer-by-layer technique,^12,13^ and compositional change of bilayer membranes by sterols,^14^ polyethylene glycol,^15^ and emulsifiers.^16^ Moreover, the same type and concentration of lipid materials with different liposome preparation methods lead to different properties, such as storage stability, encapsulation efficiency, and bilayer permeability. Therefore, many disadvantages still exist preventing further application and industrialization of food fortification with liposome structures. Thus, designing alternative types of liposomes that can make them appropriate for food formulations is still of crucial demand.

On the other hand, organogels have been considered promising types of gel structures known as novel delivery systems over the past few years.^17,18^ Organogels are self-standing, thermoreversible, and viscoelastic 3D networks which are developed by the self-assembly of gelator molecules that immobilize the continuous organic phase through hydrogen bonding, hydrophobic interactions, van der Waals forces, ionic interactions, or covalent bonding.^19,20^ The most commonly used approach for creating organogels is direct dispersion of gelator molecules in an organic liquid at temperatures above their melting points, followed by cooling to lower temperatures.^21^ Organogels exhibit inherent physical and chemical stability properties which are beneficial for longer shelf-life requirements such as delivery of bioactive agents compared to other polymer gels. Moreover, their lipid medium is well suitable for improving the bioavailability of lipid-soluble bioactive materials, and their gel network could offer a sustained release behavior and a desirable protection for the encapsulated compounds.^22^ Although there are several works on the potential of organogels for delivery applications in pharmaceutical and cosmetic applications,^23,24^ there are only a few examples of organogel applications in bulk or emulsified forms to increase the bioaccessibility of poorly water-soluble bioactive components in food systems.^25,26^ Vitamin D is a fat-soluble vitamin that can be produced in the skin by sunlight exposure. Vitamin D_3_ (cholecalciferol)_,_ as an active form of vitamin D, is essential to control calcium and phosphorus absorption in the human body.^27,28^ The deficiency of vitamin D_3_ is a worldwide concern that increases the risk of diabetes, obesity, cardiovascular diseases, and cancers.^29^ Therefore, food fortification by this micronutrient has gained increasing attention recently. Vitamins D_3_ is easily susceptible to isomerization and degradation into its inactive forms due to light, oxygen, high temperatures, and acid exposure. This fact leads to a significant reduction of vitamin D_3_ functionality and biological properties.^30^ For this purpose, several colloidal delivery systems (i.e., emulsion, solid lipid nanoparticles, nanostructured lipid carriers, and so forth) have been proposed for protecting vitamin D_3_ toward various harmful conditions, improving the oral bioavailability and delivery efficiency of vitamin D_3_, and making it soluble in aqueous systems.^31−36^ However, many disadvantages still exist, which inhibits further application and industrialization of food fortification using encapsulated vitamin D_3._ These limitations may include low-loading capacity, poor long-term stability, and loss of stability under certain ionic compositions and ingredient interactions.^37^

We hypothesized that the incorporation of an organogel network in the liposome structure would offer a highly stable delivery system of hydrophobic bioactive components. To the best of our knowledge, there are no published data on the build-up of gel structures inside bilayers for enhancing the liposome stability and encapsulation efficiency. Therefore, the aim of this work was to assess the effect of oleogelation within the lipid bilayer shell of liposomes looking for high encapsulation efficiency and improved physical stability to apply in the food formulations. In this work, nanoliposomes were prepared by an extrusion process combined with the thin-film hydration method. Vitamin D_3_ was used as a model hydrophobic bioactive agent and 3-palmitoyl-*sn*-glycerol was used as a low molecular weight organogelator due to its limited solubility in water and its manufacturing considerations.

## Materials and Methods

### Materials

l-α-Phosphatidylcholine (PC) from soybean (≥90%) was provided by Alfa Aesar (Ward Hill, Massachusetts, USA). Cholesterol (CHO) (≥99%), 3-palmitoyl-*sn*-glycerol (≥99%), vitamin D_3_ (cholecalciferol) (≥98%), sucrose (≥99.5%), glucose (≥99.5%), sodium chloride, and calcium chloride (≥99.5%) were purchased from Sigma-Aldrich (St. Louis, MI, USA) as dried powders and used without further purification. 4-Palmitamido-2,2,6,6-tetramethylpiperidine-1-oxyl (4-palmitamido-TEMPO) was also obtained from Sigma-Aldrich (St. Louis, MI, USA). All the other chemicals and reagents such as chloroform, ethanol, and methanol used were of analytical grade.

### Nanoliposome Production

Nanoliposomes were produced by a thin-film hydration method modified from Lopes et al.^38^ In brief, control liposomes were prepared by dissolving PC (300 mg) and CHO (7.5 mg) in 10 mL of chloroform. For encapsulation efficiency and in vitro digestion experiments, vitamin D_3_ (0.6 mg/L) was added to the mixture. For EPR experiments, a spin probe was added to the solution at a concentration of 1%. Then, the organic solvent was removed using a rotary evaporator at a temperature of 60 °C until a thin lipid film was formed. This process was followed by 10 min of vacuum treatment at controlled reduced pressure and under a nitrogen stream for 2 min to make sure that no trace of organic solvent remained. For the development of an organogel network within the bilayer shell of nanoliposomes, soybean oil (8.5 mg) and 3-palmitoyl-*sn*-glycerol (1.5 mg) were added to the mixture, and the other processes were the same as the preparation of control liposomes. To keep the properties of the liposome intact and prevent the oxidation of phospholipids until their use, they were stored in a freezer at a temperature of −80 °C. Subsequently, the lipid bilayers were hydrated by adding 10 mL of Milli-Q water and then a rotary evaporator (without vacuum) was used to form multilamellar large vesicles (MLVs). To obtain homogeneous small unilamellar vesicles (SUVs), the sample was subjected to extrusion by passing the suspension through the 400, 200, and 100 nm polycarbonate membranes sequentially. After enough extrusion steps through the membrane with the help of a Thermobarrel Extruder (Northern Lipids, Vancouver, Canada) under nitrogen pressures up to 55 bar at 60 °C, a normal unimodal distribution was obtained. The obtained nanoliposomes were stored at 4 °C prior to further use.

### Physical Properties of the Bulk Organogel

To determine the mechanical and viscoelastic properties of the organogel developed in the lipid bilayer of nanoliposomes, a sample of organogel was prepared by dissolving 3-palmitoyl-*sn*-glycerol (15% w/w) in soybean oil at 60 °C, followed by cooling to room temperature. A texture analyzer (Texture Analyzer, TA Plus, Stable Microsystems, Surrey, UK) with a load cell of 30 kg and a cylindrical probe was used to determine the mechanical properties of the organogel after 24 h of storage at 25 °C, as described by Giacomozzi et al.^39^ with some modifications. Viscosity and dynamic rheological measurements were also performed using an MCR 302 controlled stress/strain rheometer (Anton Paar, Graz, Austria) equipped with a parallel plate geometry.^26^ The strain sweep test from 0.002 to 1% at a constant frequency of 1 Hz was performed to determine the linear viscoelastic region (LVR) of the organogel. Then, the frequency sweep (0.01–10 Hz) and the temperature ramp from 5 to 80 °C at the rate of 2 °C/min and 1 Hz frequency were carried out inside the LVR region.

### Particle Size

The particle size and polydispersity index (PDI) were obtained by means of a dynamic light scattering (DLS) device (Zeta Sizer Nano, ZS-90 Malvern Instruments Ltd., UK). The experiments were performed at 25 °C in quasi-backscattering configuration (scattering angle, θ = 173°) using the radiation from the red line of a He–Ne laser (wavelength, λ = 632 nm), using a refractive index of 1.459. Samples were diluted at 1:50 ratio in Milli-Q water, and the obtained results were reported as intensity-weighted.

### Zeta Potential Measurement

The zeta potential (ZP) experiment was carried out using the laser Doppler velocimetry (LDV) technique in a Zetasizer Nano device (ZS-90 Malvern Instruments Ltd., UK) that measures the electrophoretic mobility of the sample from the speed of the particles. A DTS 1060 cuvette with a polycarbonate capillary was used, and the measurements were made at a constant temperature of 25 °C after dilution of the liposomes in Milli-Q water (ratio, 1:50).

### Stability Measurement

#### Storage Stability

The mean vesicle size, PDI, and ZP of empty nanoliposomes were determined at scheduled time intervals (day 1, 6, 18, 36, 48, 60, and 90) during a 3 month storage period at 4 °C.^40−42^ Their physical stability was also monitored through observation for any visual instabilities, such as fusion and aggregation.

#### Salt and Sugar Stability

The stability of nanoliposomes against food ingredients or additives, such as salts and sugars, is a crucial aspect for their food applications as delivery systems. For this reason, 0–20% (w/v) sucrose and glucose, as well as 0–5% chloride salts of sodium, potassium, and calcium ions, were explored for their effects on vesicle size and macroscopic stability.^5^

#### Lipid Bilayer Fluidity

In order to study the membrane fluidity, nanoliposome samples were labeled using the spin label 4-palmitamido-TEMPO, which is located in the middle part of the bilayer. EPR spectra were recorded in the temperature range interval from 15 to 60 °C and at the X-band microwave frequency of 9.85 GHz using a Bruker EMX-Plus spectrometer (Germany) with temperature control by nitrogen circulation. *W*_0_, in Gauss (G), and heights of the mid- and high-field lines, *h*_0_ and h_–1_, respectively, were obtained from each absorption spectrum. The rotational correlation time (Τ_R_) was calculated according to^43^
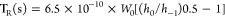


#### Morphological Studies

Transmission electron microscopy (TEM) was used for determining the nanoliposome microstructure. One drop (10 μL) of each sample was deposited on a carbon-coated copper grid and allowed to dry for 60 s. Then, the grids were stained with a drop of 2% uranyl acetate solution for 50 s and the excess stain was wicked away with a piece of filter paper. The air-dried samples were observed using a TEM (Jeol JEM-1400, Jeol Ltd., Tokyo, Japan) at an acceleration voltage of 120 kV.

#### Encapsulation Efficiency and Loading Capacity

Vitamin D_3_ is a hydrophobic molecule, hence the concept was that during encapsulation in nanoliposomes, it would be deposited within the lipid bilayer. To measure the encapsulation efficiency (EE, %), a certain amount of each kind of loaded nanoliposomes was washed three times with phosphate-buffered saline (PBS) to make sure that free vitamin D_3_ was not detected in the supernatant_._ The remaining pellets (loaded liposomes) were dissolved in ethanol to promote liposomal membrane lysis and then the suspension was studied by UV spectrophotometry using an UV–vis spectrophotometer (Jasco, V-630, Japan) at 264 nm. Unloaded nanoliposomes were also investigated as controls.^33^ The respective EE and loading capacity (%) were calculated using the following equations
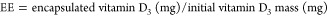

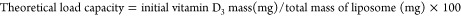

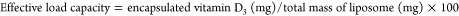


#### Statistical Analysis

Each experiment was performed at least in triplicate. Statistical analysis was conducted using SAS software (ver. 9.1.3, SAS Institute Inc., Cary, NC, US). Analysis of variance was performed using one-way analysis of variance (ANOVA). The results are expressed as mean values ± SD. The significance level was set at *P* ≤ 0.05.

## Results and Discussion

### Mechanical and Viscoelastic Properties of the Bulk Organogel

**Figure 1:**
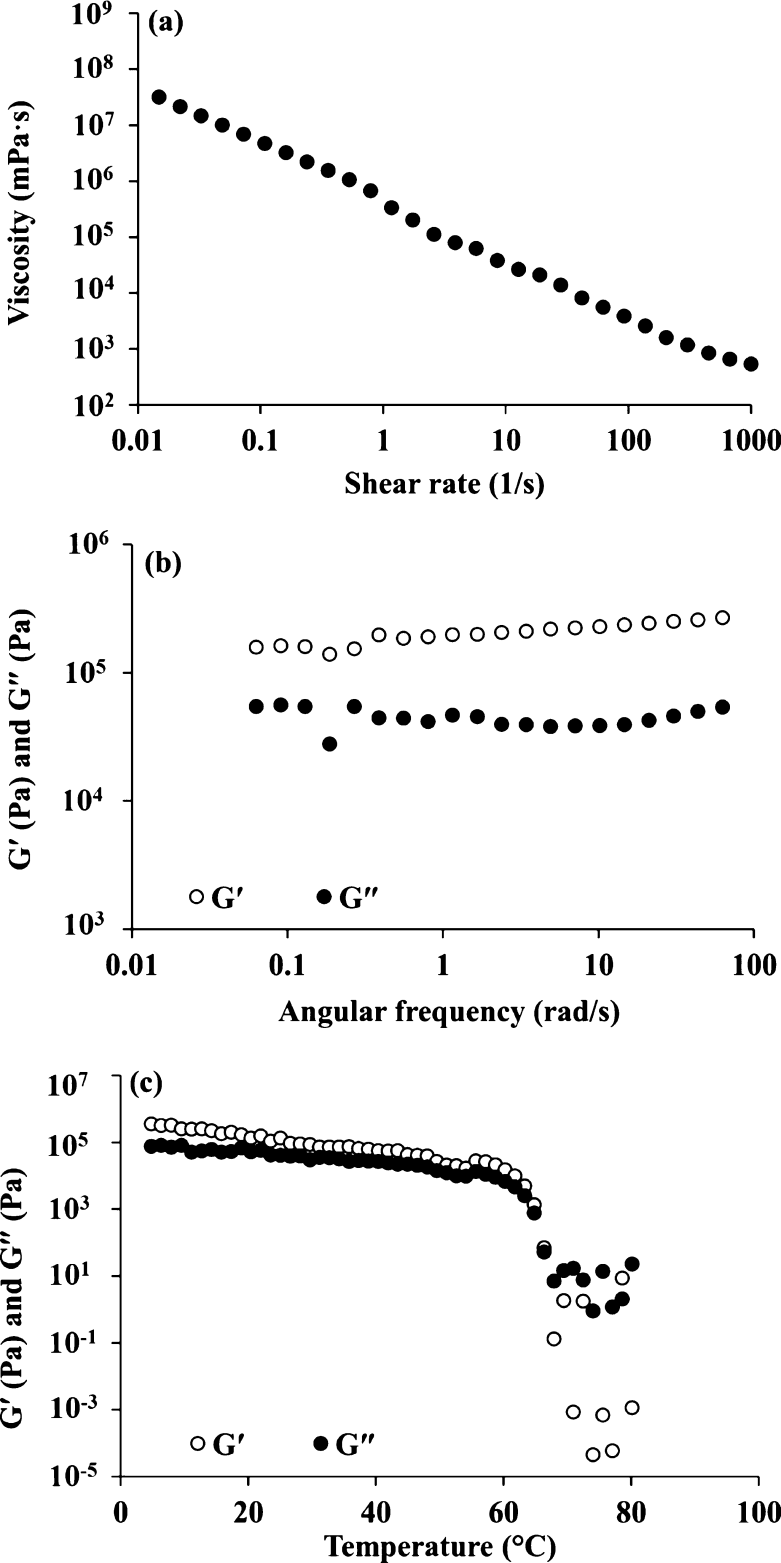
(a) Evaluation of the apparent viscosity with the shear rate and (b,c) changes in the storage modulus (*G*′) and loss modulus (*G*″) as a function of frequency sweep and temperature ramp test, respectively, for the organogel at 25 °C.

According to the texture studies, hardness, adhesiveness, and cohesiveness of the organogel network were 141.32 ± 2.83 g, 684.99 g.s, and 0.38, respectively. As shown in Figure 1a, *G*′ values were always higher than the *G*″ values in the whole frequency range applied, and the plateau region of the mechanical spectrum is always noticed in the LVR region, indicating the predominant elastic gel-like behavior of the organogel. This finding was in accordance with the result reported by Rocha et al.,^44^ who found such elastic behavior for the organogel developed from sugarcane or candelilla wax in soybean oil. According to the temperature ramp test, at temperatures lower than 60 °C, *G*′ was greater than *G*″, which confirmed the presence of a strong gel network. Increasing the temperature led to noticeably changed viscoelastic properties as the loss modulus was higher than the storage modulus, indicating the predominant viscous behavior of the sample due to the melting of the three-dimensional network organogel. Moreover, the evolution of viscosity with shear rate (Figure 1c) showed the pronounced non-Newtonian shear-thinning nature.^45^ The high values of viscosity confirmed the successful development of a stable gel structure as a consequence of the intermolecular junction zones through non-covalent interactions.

### Build-Up of the Supramolecular 3-D Gel Structure within the Lipid Bilayer Shell of Nanoliposomes

**Figure 2:**
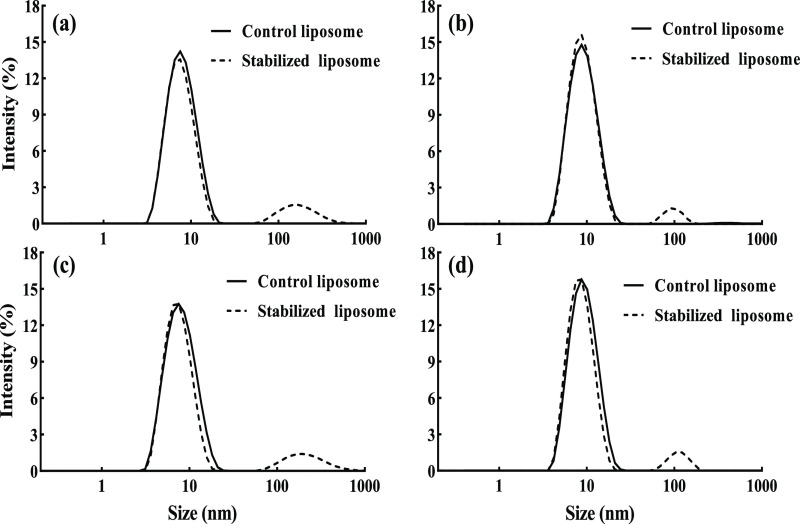
Particle size distribution of the control liposome and that stabilized with the 3D organogel network within the bilayer shell as affected by (a) 20 and (b) 30 mM Triton X-100 immediately after exposure and by (c) 20 and (d) 30 mM Triton X-100 3 days after exposure.

The aim of this work was to build-up a 3-D supramolecular gel structure within the lipid bilayers, generating a nanoliposome with a gelled shell, which can likely improve the loading and prolonged stability of loaded liposomes. It is generally accepted that the exposure of liposomes to detergents, close to the critical micellar concentration (CMC), leads to disruption, instantaneous destabilization of liposomes, and subsequent leakage and loss of encapsulated materials into the water phase.^46^ Our expectation was that the organogel structure developed within the bilayers remained stable upon detergent treatment, leading to a complete or partial stability of the lipid membranes. In order to determine this hypothesis, we added Triton X-100 at the concentrations of 20 and 30 mM into the nanoliposome dispersions and then monitored the following changes in particle size immediately and about 3 days after exposure. Regardless of the Triton concentration and exposure time (Figure 2), the control nanoliposome dispersion was not able to withstand the intercalation of detergent into the lipid bilayer, resulting in a complete disturbance of the lipid bilayers and a noticeable decrease in the vesicle diameter from the initial size (102.5 nm). Therefore, upon destabilization of control liposomes, only one particle size population around 7–8 nm, which corresponded to Triton X-100 micelles filled with phospholipids, was observed at both Triton concentration and exposure time studied, as shown in Figure 2. These changes imply that the addition of detergent promotes opening up and fragmentation of the vesicles, leading to the formation of Triton–phospholipid micelles and finally the complete solubilization of the bilayers by the detergent micelles.^47^ By addition of 20 mM Triton X-100 to the nanoliposome sample with a gelled shell structure, both small (∼10–20 nm) and intermediate (60–100 nm) particles were observed (Figure 2a). The small particles related to the mixed lipid–detergent micelles and the intermediate size represented unsolubilized membrane vesicles. This trend was also observed at a higher concentration (30 mM) of Triton X-100 (Figure 2b). Regardless of the Triton X-100 concentration, the intermediate vesicle population still remained stable after 3 days of exposure in the liposome sample stabilized by the organogel structure within the bilayer, as shown in Figure 2c,d. These results confirmed the higher prolonged stability of liposomes in the presence of a gelled lipid shell. This effect can be explained by the fact that the organogel network makes a strong scaffold in the lipid bilayer, which provides more stability to nanoliposomes against fragmentation compared to the control ones toward detergent digestion.

### Characterization and Storage Stability of Nanoliposomes

**Figure 3:**
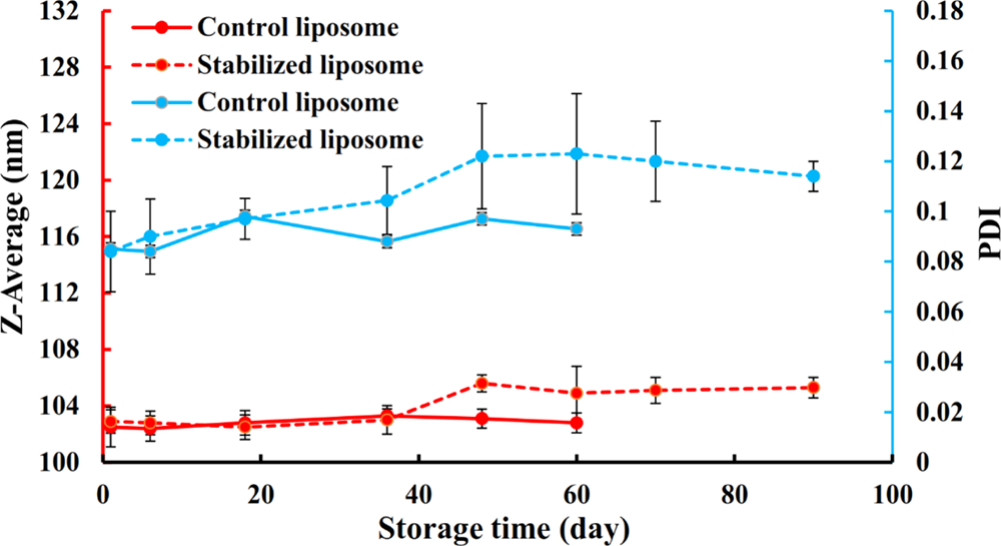
Z-average (nm) and PDI of the control liposome and that stabilized with the 3D organogel network within the bilayer shell stored for 90 days at 4 °C.

The DLS technique evaluates the apparent hydrodynamic diameter of particles in a colloidal system. Figure 3 shows the intensity-weighted diameter and the PDI of empty nanoliposomes. Fresh control samples and those stabilized with a gelled lipid shell showed mean diameters around 102 nm. Thus, the presence of the organogel network within the bilayer shell did not significantly (*P* ≥ 0.05) affect the mean hydrodynamic diameter of fresh samples. The PDI values, which determine the degree of size homogeneity, were 0.085 and 0.084 for the control sample and the sample with a gelled lipid bilayer shell, respectively. These small values of PDI (<0.3) indicate a very narrow size population of nanoliposomes^48^ in both fresh samples. These results are similar to those previously reported by Kakami et al.^49^ and Yusuf and Casey,^50^ who reported 128 and 140 nm for nanoliposomes obtained by the extrusion process, respectively.

**Figure 4:**
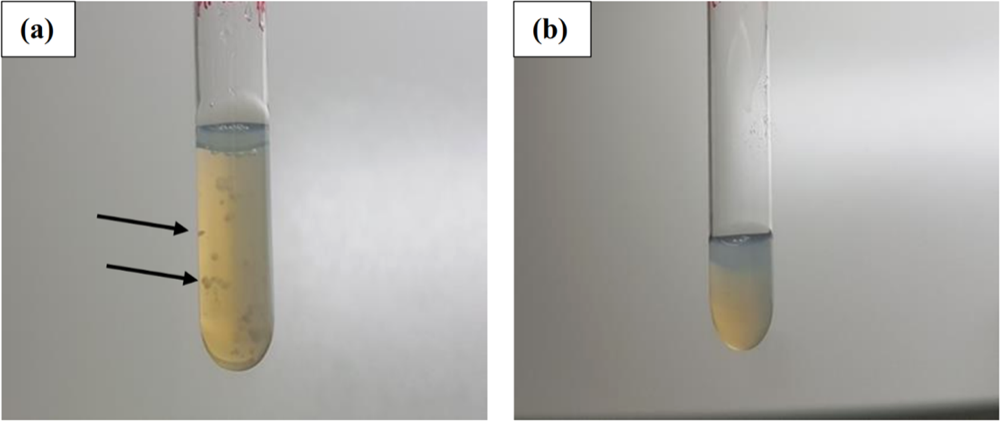
Observation of physical stability of (a) the control liposome and (b) that stabilized with the 3D organogel network within the bilayer shell stored for 90 days at 4 °C. Black arrows indicate agglomerated and flocculated vesicles.

According to Figure 3, the storage time did not significantly affect the size and PDI values for the control sample up to 2 months of storage. However, the clear evidence of agglomerated and flocculated particles was observed from day 64 in control liposomes (Figure 4), which made sampling afterward impossible as the measurement of such samples by DLS does not provide reliable results due to the high level of inhomogeneity. This visual instability was in good agreement with zeta potential results that were studied in detail below. In the case of nanoliposomes with a gelled bilayer shell, the particle size and PDI did not change during the first month of storage. Although the particle size and PDI exhibited slight increases from the beginning after 38 days, this sample remained physically stable for more than 3 months with no color change and any obvious agglomeration or flocculation. The small value of PDI at the end of storage time (0.114) also represented a monodispersed distribution and high level of homogeneity, hence excellent liposome stability (Figure 3).

**Figure 5:**
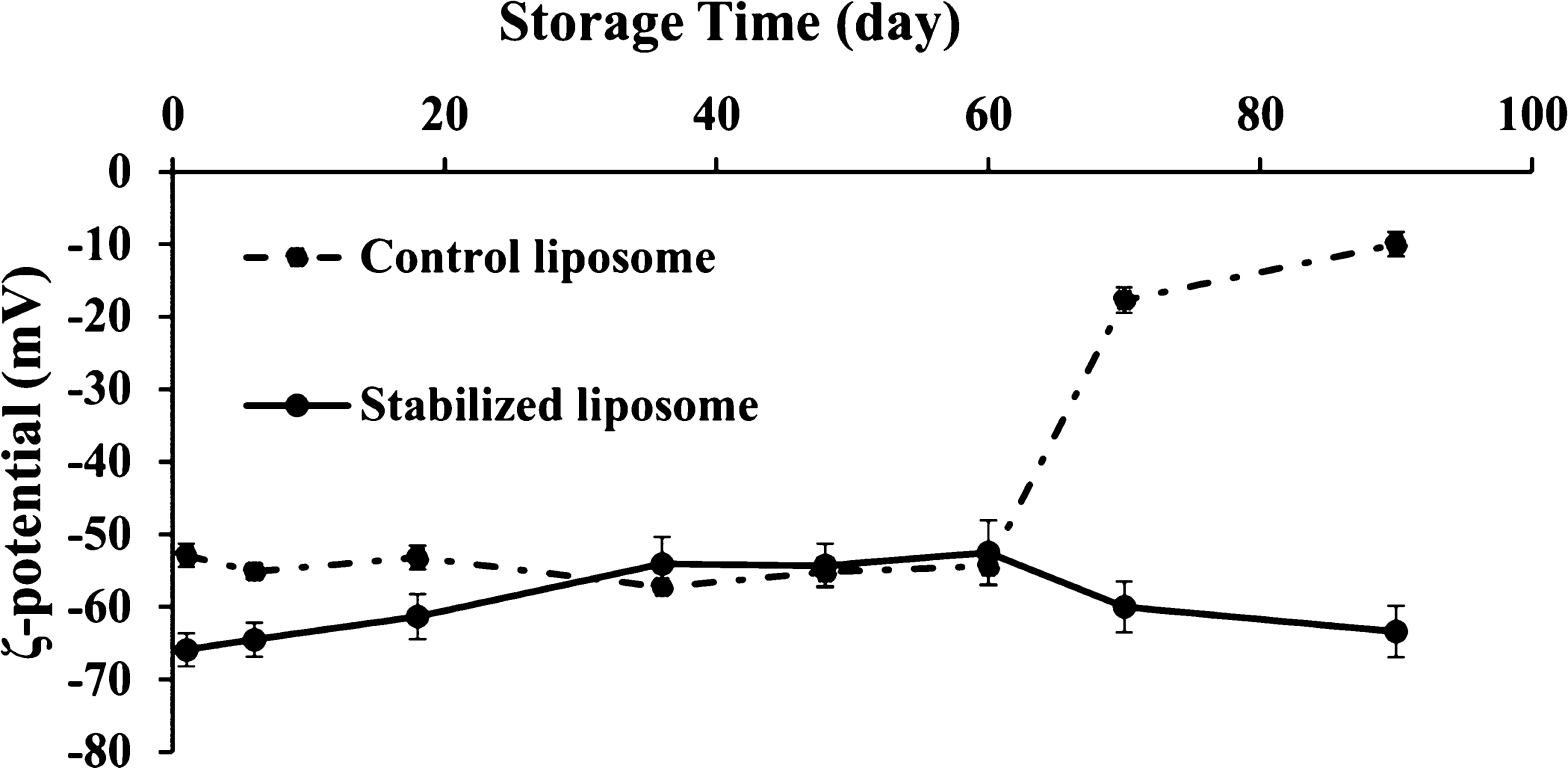
Zeta potential of the control liposome and that stabilized with the 3D organogel network within the bilayer shell stored for 90 days at 4 °C.

Zeta potential is a significant indicator to predict the physical stability of colloidal suspensions.^10^ In this regard, the range of −30 to +30 mV shows the instability of a colloid, and the degree of instability rises when the zeta potential approaches zero.^8^ The potential values of fresh control samples and those with the gelled shell structure were −52 and −65 mV, respectively (Figure 5). This can be explained considering that the PC used in this work was negatively charged in its original state. Moreover, the possible presence of FFAs in the organogelator and soybean oil might contribute to the observed more negative charge in nanoliposomes stabilized by the organogel network within the lipid bilayer shell.^26^ These high negative surface charges were indicative of high strong stability of freshly prepared liposomes. As clearly seen from Figure 5, the electronegative zeta potential values remained relatively constant for the control sample up to 60 days. However, during the next 10 days, the zeta potential showed a sharp decrease to −17 mV and then reached −9 mV at the end of storage (90 days). This trend to neutralization led to the agglomeration of large particles, followed by breakdown of the system as discussed previously. On the contrary, the zeta potential of liposomes stabilized with the gel structure within the lipid bilayer remained highly negative (−63 mV) during the entire storage time (Figure 5), suggesting high electrostatic repulsion and excellent stability of vesicle structures. Our findings proved that the development of a supramolecular organogel structure between lipid bilayers can improve the long-term stability of liposomes, which was also in line with visual observation and particle size measurement.

### Salt and Sugar Stability of Nanoliposomes

**Figure 6:**
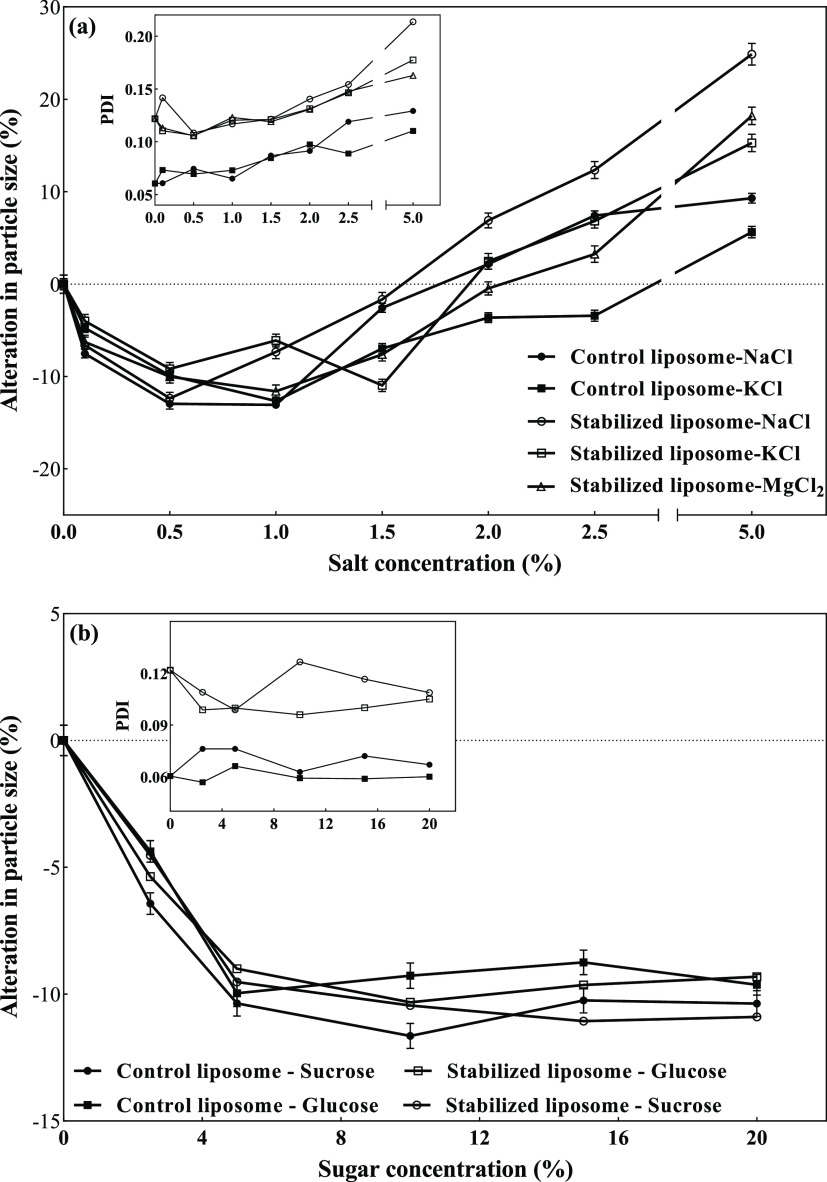
Alteration in the particle size of the control liposome and that stabilized with the 3D organogel network within the bilayer shell in the presence of increasing concentrations of different (a) salts and (b) sugars.

One of the main limitations of liposomes as a carrier in food applications is their poor stability and relatively high semi-permeability of membranes due to osmotic pressure toward food components or additives such as sugars or salt. Thus, the effect of different salts and sugars on nanoliposome suspension stability was examined by incubating them at room temperature for 120 min, as shown in Figure 6a,b. In control liposomes, the presence of 0.1% monovalent potassium and sodium cation salts already led to small alterations in liposomal sizes (<10%), which increased up to 1.5% salt concentration (Figure 6a). A similar trend was observed in the sample containing an organogel structure between the bilayer shell but with a lower size reduction. This small decrease in vesicle size by addition of NaCl or KCl is in agreement with the findings of Frenzel and Steffen-Heins.^5^ There are two hypotheses to explain the salt effect. First, the mechanism of cation adsorption onto the surface of bilayer, which creates a change in the head group charge, leading to a change in the curvature of bilayer due to the electrostatic interactions and therefore the reduction of liposome size.^5,51^ Second, due to the increase of osmotic force gradient across liposome membranes, some of the water molecules are transferred from the inner aqueous phase to the outer aqueous phase to adjust the external excess ion concentration, resulting in liposome shrinkage and hence a decrease in their size.^9,52^ In addition, the head group dehydration in the presence of low concentration of salt ions resulted in an imbalance of hydrophobic and electrostatic attractions and interfacial tension, which were responsible for system membrane stability, leading to the squeeze of alkyl chains and a decrease of liposome size.^5^ For salt concentrations higher than 2%, there was an increase in the particle diameter (Figure 6a), indicating some liposome aggregation due to screening of the electrostatic repulsion between them by the cations.^53^ Other researchers have also observed a similar phenomenon with other types of liposomes.^51,54^ It should be noted that there was no significant size increase and aggregation to produce visible clusters. Therefore, both liposomes remained stable in all ranges of monovalent salt concentrations. However, after 1 day of storage at 4 °C, a white sediment at the bottom of control samples and a slight increase in turbidity of the sample stabilized with an organogel structure between the bilayer were observed. These effects may indicate relatively better stability of the latter sample due to the lower permeability of ions through the membrane stabilized with an organogel network. As shown in Figure 6a, even small concentrations of magnesium cations resulted in immediate breakdown of control liposomes. In contrast, the liposome sample stabilized with an organogel network between the bilayer shell remained completely stable in the presence of magnesium salts up to 5%, which clearly indicated that the gel network avoided divalent cation interaction with phosphate residues within bilayers. This property permits the application of this novel structure of liposomes in dairy products containing high divalent ion concentrations.

In the presence of sugars, the changes in liposome size were similar for both samples. Sucrose and glucose reduced the particle size around 10%, which remained constant up to 20% sugar concentration. This phenomenon was related to the osmotic activity of sugars, as previously discussed for salts.

### Lipid Bilayer Fluidity

**Figure 7:**
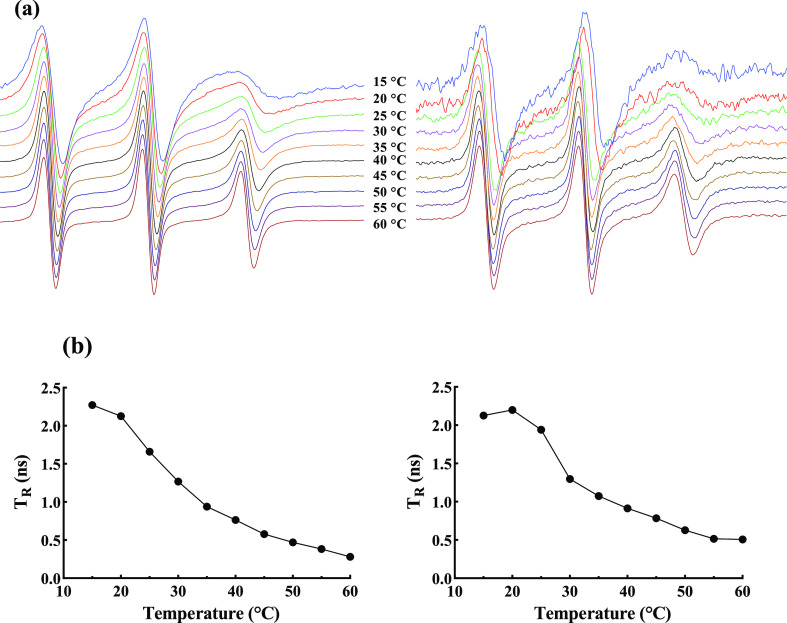
(a) Temperature dependence of the ESR spectra of 4-palmitamido-TEMPO reconstituted in bilayers and (b) rotational correlation time (T_R_) as a function of temperature of (left) the control liposome and (right) that stabilized with the 3D organogel network within the bilayer shell. Total spectral width = 60 Gauss.

The lipid bilayer fluidity of liposomes describes the molecular ordering and dynamics of phospholipid alkyl chains in the membrane, which are generally dependent on its composition.^55^ EPR is a useful spectroscopic technique for detecting changes in the membrane fluidity of liposomes.^56,57^ In EPR, there is a direct connection between the spin label mobility and the viscosity of its surrounded area to explain the gel (less mobility) or liquid–crystalline (high mobility) phases.^58^ The solubilization of the spin probe did not change the zeta potential and the particle size of liposomes (data not shown). The experimental ESR spectra of 4-palmitamido-TEMPO at different temperatures, ranging between 15 and 60 °C, are shown in Figure 7a. In the presence of a gelled bilayer shell in nanoliposomes (Figure 7a right side), much broader and anisotropic ESR spectra were obtained, demonstrating a large reduction in the rotational motion of the probe. Moreover, the differences between two extreme positions increased in a similar way as the temperature decreased, implying that the presence of the organogel network in the bilayers restricted the phospholipid chain mobility in membranes.

Figure 7b presents the rotational correlation time which is sensitive to the rotational motional freedom close to the polar head groups and hence the viscosity of the hydrophobic region of the bilayer. Since T_R_ is inversely related to the fluidity, a significant decrease in this parameter was reported for both samples by increasing the temperature (Figure 7b). As previously reported, the membrane fluidity of liposomes similarly increased when the temperature rises.^5,59^ In addition, control samples showed a significant increase in the mobility at temperatures above phase transitions of PC (35 °C). As shown in Figure 7b (right side), liposome samples stabilized with the organogel network between bilayers had a higher T_R_, suggesting the higher rigidity and a slower rotational motion of the alkyl chains in the hydrophobic part of the bilayer than the control sample. This effect is related to the formation of a 3D gel network within the bilayer shell by hydrogen bonding between the free hydroxyl groups of 3-palmitoyl-*sn*-glycerol with the ester carbonyl group of soybean oil, leading to the tight packing of phospholipid chains in the gel phase.

### Morphological Studies

**Figure 8:**
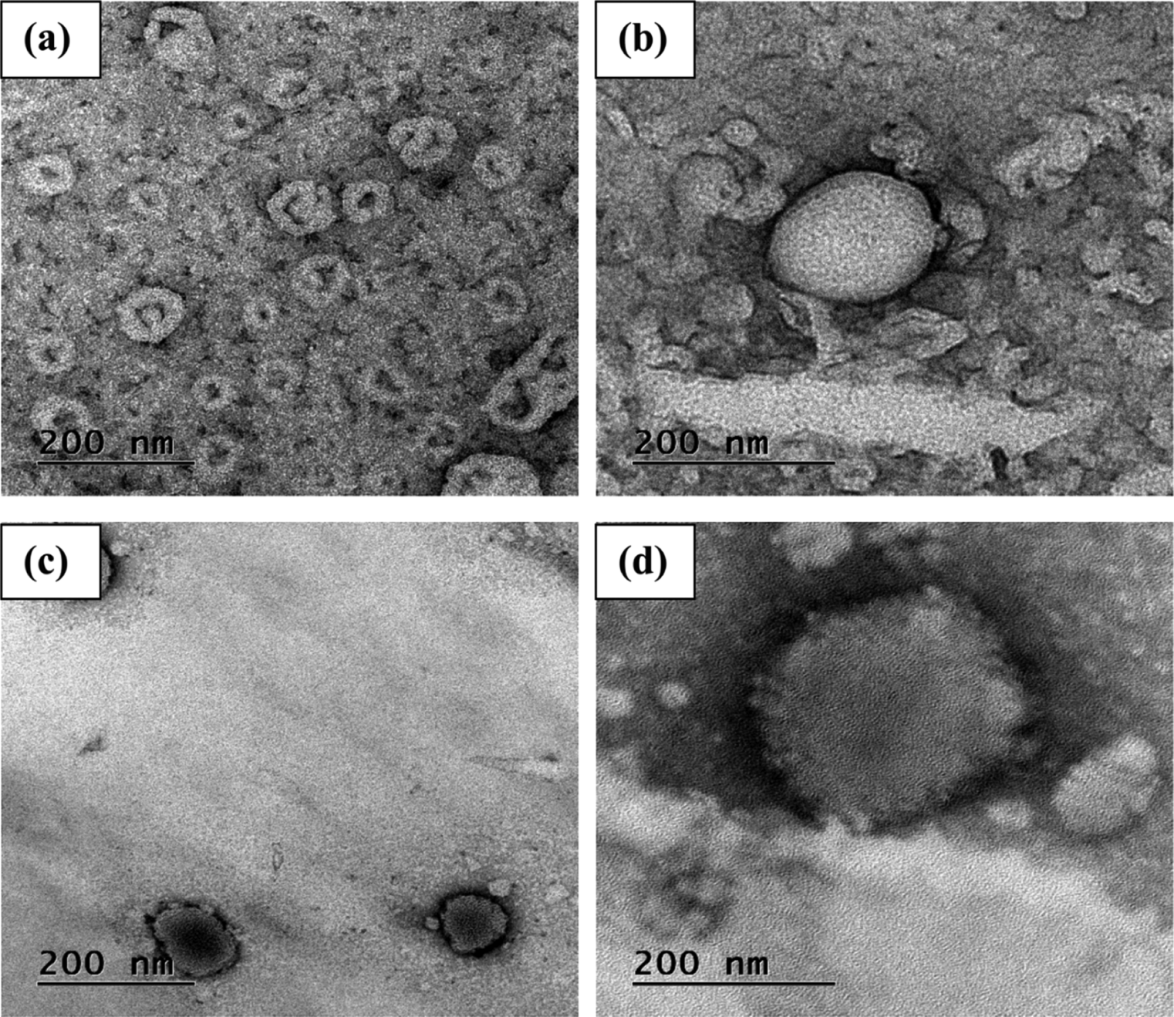
TEM morphology of the control liposome (a) after preparation and (b) after storage and that stabilized with the 3D organogel network within the bilayer shell (c) after preparation and (d) after storage.

As TEM observations need very low sampling capacity, the general trend cannot be easily obtained, but it can provide useful evidence about the morphology, size, integrity, and homogeneity of liposomes which are important for manipulating the liposome characteristics for delivery applications.^60^ The microstructures of the nanoliposome suspensions are presented in Figure 8. In fresh control samples, spherical- to elliptical-shaped particles with a rather low size distribution were seen. However, there was evidence of vesicles’ tendency to aggregate (Figure 8a). The spherical structure and well-separated unilamellar vesicles were also visually observed in fresh samples stabilized with the organogel network between the lipid bilayer (Figure 8b). It seems that the presence of a gel structure may have altered the optimum curvature of the lipid bilayer, thereby favoring the formation of equally stable vesicles. According to TEM images before staining (date not shown), the bilayer thickness values of liposomes were 3.2 and 3.6 nm for the control sample and the stabilized one with an organogel structure between the lipid bilayer shell, respectively. Therefore, the development of a 3D supramolecular gel network within the bilayer can increase the membrane thickness while keeping the vesicle size constant. In TEM micrographs of control liposomes after 3 months of storage (Figure 8c), the formation of agglomerated particles and the evidence of membrane fusion were observed which are likely be attributed to the high fluidity of the membranes. As clearly seen from Figure 8d, no significant differences were observed over time in the microstructure of liposomes stabilized with an organogel network between the lipid bilayer, suggesting its longer storage stability than control liposomes.

In brief, TEM results confirmed the results of particle size measurements conducted by DLS. However, there were some differences in the liposome size determined by TEM and DLS, which can be attributed to the differences in the sample preparation methods (e.g., staining and drying), as well as to different physical principles of the two techniques,^26,61^ but the overall trends were similar.

### Encapsulation Efficiency and Loading Capacity

The results of nanoliposome EE and LC are reported in Table 1. The EE of vitamin D_3_ was 36 and 71% for the control sample and those incorporated with the gel network between the bilayer, respectively. These effects may have been a result of higher fluidity of membranes in the control sample which led to easier membrane fusion and vitamin D_3_ leakage. In contrast, the more ordered structures of lipid membranes with higher rigidity in the presence of an organogel network between the bilayer shell contributed to an improved encapsulation of vitamin D_3_ within the hydrophobic area of liposomes. The high efficiency of the lipid gelled structure to encapsulate the bioactive component was also reported previously in colloidal dispersion.^26^ Compared to the previous report on vitamin D_3_ encapsulation in liposomes using a thin-film hydration–sonication method at the same concentrations of PC and CHO, which reported an EE around 57%,^33^ the obtained EE in the present work for the novel structure of liposomes stabilized with the organogel network was 1.25-fold greater. Although Mohammadi et al.^62^ reported more than 93% EE for vitamin D_3_ in nanoliposomes by the thin-film hydration–sonication method, which is comparable to that obtained in this study, their fabrication method included three stages including thin-film hydration, homogenization, and sonication, and the obtained nanoliposomes had lower physical stability during storage.

**Table 1:** Encapsulation Efficiency and Loading Capacity of Vitamin D3 in the Control Liposome and That Stabilized with the 3D Organogel Network within the Bilayer Shell

	control liposome	stabilized liposome
encapsulation efficiency (%)	36.85 ± 3.67^a^^,b^	71.54 ± 17.20^a^
theoretical loading capacity (%)	0.98	0.94
effective loading achieved (%)	0.36 ± 0.04^b^	0.68 ± 0.16^a^

a Results are presented as mean ± standard deviation. Different uppercase letters within a row are significantly different (*P* < 0.05).

Moreover, the high effective load (0.68%) of vitamin D_3_ in liposomes stabilized with the organogel structure between the bilayer (Table 1) also confirmed the significant potential of the 3D organogel network to protect and embed the hydrophobic molecules. In fact, the presence of a gel network between the bilayer shell resulted in less fluid membranes and higher molecular packing density, leading to an increase in membrane thickness and loading capacity. These results showed the promising potential of stabilized nanoliposomes with an organogel shell structure to encapsulate hydrophobic bioactive materials with high encapsulation efficiency, which was obtained by complex interactions including hydrogen bonding and hydrophobic interactions.^63^ Consequently, this approach of formulating food-grade phospholipid nanostructures could be potentially valuable for a wide range of applications in the efficient delivery of food and pharmaceutical bioactive compounds.

## Abbreviations

CMC: critical micellar concentration
CHO: cholesterol
DLS: dynamic light scattering
EE: encapsulation efficiency
EPR: electron paramagnetic resonance
FFAs: free fatty acids
LC: loading capacity
LDV: laser Doppler velocimetry
LVR: linear viscoelastic region
MLVs: multilamellar large vesicles
PBS: phosphate-buffered saline
PC: l-α-phosphatidylcholine
PDI: polydispersity index
SUVs: small unilamellar vesicles
TEM: transmission electron microscopy
ZP: zeta potential

## References

[ref1] Toro-Uribe S.; López-Giraldo L. J.; Decker E. A. Relationship between the physiochemical properties of cocoa procyanidins and their ability to inhibit lipid oxidation in liposomes. J. Agric. Food Chem. 2018, 66, 4490–4502. 10.1021/acs.jafc.8b01074. 29649362

[ref2] Peng H.; Chen S.; Luo M.; Ning F.; Zhu X.; Xiong H. Preparation and Self-Assembly Mechanism of Bovine Serum Albumin–Citrus Peel Pectin Conjugated Hydrogel: A Potential Delivery System for Vitamin C. J. Agric. Food Chem. 2016, 64, 7377–7384. 10.1021/acs.jafc.6b02966. 27622937

[ref3] Smith A. M.; Jaime-Fonseca M. R.; Grover L. M.; Bakalis S. Alginate-loaded liposomes can protect encapsulated alkaline phosphatase functionality when exposed to gastric pH. J. Agric. Food Chem. 2010, 58, 4719–4724. 10.1021/jf904466p. 20225816

[ref4] Ramalho M. J.; Coelho M. A.; Pereira M. C. Nanoparticles for delivery of vitamin D: Challenges and opportunities. In A Critical Evaluation of Vitamin D—Clinical Overview; Gowder S., Ed. 2017; p 11.

[ref5] Frenzel M.; Steffen-Heins A. Whey protein coating increases bilayer rigidity and stability of liposomes in food-like matrices. Food Chem. 2015, 173, 1090–1099. 10.1016/j.foodchem.2014.10.076. 25466129

[ref6] Gómez-Mascaraque L. G.; Casagrande Sipoli C.; de La Torre L. G.; López-Rubio A. Microencapsulation structures based on protein-coated liposomes obtained through electrospraying for the stabilization and improved bioaccessibility of curcumin. Food Chem. 2017, 233, 343–350. 10.1016/j.foodchem.2017.04.133. 28530583

[ref7] Zhou F.; Xu T.; Zhao Y.; Song H.; Zhang L.; Wu X.; Lu B. Chitosan-coated liposomes as delivery systems for improving the stability and oral bioavailability of acteoside. Food Hydrocolloids 2018, 83, 17–24. 10.1016/j.foodhyd.2018.04.040.

[ref8] Guner S.; Oztop M. H. Food grade liposome systems: Effect of solvent, homogenization types and storage conditions on oxidative and physical stability.. Colloids Surf., A 2017, 513, 468–478. 10.1016/j.colsurfa.2016.11.022.

[ref9] Frenzel M.; Krolak E.; Wagner A. E.; Steffen-Heins A. Physicochemical properties of WPI coated liposomes serving as stable transporters in a real food matrix. LWT - Food Sci. Technol. 2015, 63, 527–534. 10.1016/j.lwt.2015.03.055.

[ref10] Li R.; Zhang L.-Y.; Li Z.-J.; Xue C.-H.; Dong P.; Huang Q.-R.; Wang Y.-M.; Zhang T.-T. Characterization and absorption kinetics of a novel multifunctional nanoliposome stabilized by sea cucumber saponins instead of cholesterol. J. Agric. Food Chem. 2019, 68, 642–651. 10.1021/acs.jafc.9b06460. 31830780

[ref11] Takagi I.; Shimizu H.; Yotsuyanagi T. Application of alginate gel as a vehicle for liposomes. I. Factors affecting the loading of drug-containing liposomes and drug release. Chem. Pharm. Bull. 1996, 44, 1941–1947. 10.1248/cpb.44.1941. 8904824

[ref12] Li Z.-l.; Peng S.-f.; Chen X.; Zhu Y.-q.; Zou L.-q.; Liu W.; Liu C.-m. Pluronics modified liposomes for curcumin encapsulation: Sustained release, stability and bioaccessibility. Food Res. Int. 2018, 108, 246–253. 10.1016/j.foodres.2018.03.048. 29735054

[ref13] Correa S.; Boehnke N.; Deiss-Yehiely E.; Hammond P. T. Solution conditions tune and optimize loading of therapeutic polyelectrolytes into layer-by-layer functionalized liposomes. ACS Nano 2019, 13, 5623–5634. 10.1021/acsnano.9b00792. 30986034 PMC6980385

[ref14] Mannock D. A.; Lewis R. N. A. H.; McMullen T. P. W.; McElhaney R. N. The effect of variations in phospholipid and sterol structure on the nature of lipid–sterol interactions in lipid bilayer model membranes. Chem. Phys. Lipids 2010, 163, 403–448. 10.1016/j.chemphyslip.2010.03.011. 20371224

[ref15] Abe K.; Higashi K.; Watabe K.; Kobayashi A.; Limwikrant W.; Yamamoto K.; Moribe K. Effects of the PEG molecular weight of a PEG-lipid and cholesterol on PEG chain flexibility on liposome surfaces. Colloids Surf., A 2015, 474, 63–70. 10.1016/j.colsurfa.2015.03.006.

[ref16] Tai K.; He X.; Yuan X.; Meng K.; Gao Y.; Yuan F. A comparison of physicochemical and functional properties of icaritin-loaded liposomes based on different surfactants. Colloids Surf., A 2017, 518, 218–231. 10.1016/j.colsurfa.2017.01.019.

[ref17] Sagiri S. S.; Kasiviswanathan U.; Shaw G. S.; Singh M.; Anis A.; Pal K. Effect of sorbitan monostearate concentration on the thermal, mechanical and drug release properties of oleogels. Korean J. Chem. Eng. 2016, 33, 1720–1727. 10.1007/s11814-015-0295-4.

[ref18] Qiu C.; Huang Y.; Li A.; Ma D.; Wang Y. Fabrication and characterization of oleogel stabilized by gelatin-polyphenol-polysaccharides nanocomplexes. J. Agric. Food Chem. 2018, 66, 13243–13252. 10.1021/acs.jafc.8b02039. 30485099

[ref19] O’Sullivan C. M.; Barbut S.; Marangoni A. G. Edible oleogels for the oral delivery of lipid soluble molecules: composition and structural design considerations.. Trends Food Sci. Technol. 2016, 57, 59–73. 10.1016/j.tifs.2016.08.018.

[ref20] Jadhav S. R.; Hwang H.; Huang Q.; John G. Medium-chain sugar amphiphiles: a new family of healthy vegetable oil structuring agents. J. Agric. Food Chem. 2013, 61, 12005–12011. 10.1021/jf401987a. 24236574

[ref21] Patel A. R.; Dewettinck K. Edible oil structuring: an overview and recent updates. Food Sci. Technol. Bull. Funct. Foods 2016, 7, 20–29. 10.1039/c5fo01006c. 26415120

[ref22] Martinez R. M.; Rosado C.; Velasco M. V. R.; Lannes S. C. S.; Baby A. R. Main features and applications of organogels in cosmetics. Int. J. Cosmet. Sci. 2019, 41, 109–117. 10.1111/ics.12519. 30994939

[ref23] Dhal S.; Mohanty A.; Yadav I.; Uvanesh K.; Kulanthaivel S.; Banerjee I.; Pal K.; Giri S. Magnetic nanoparticle incorporated oleogel as iontophoretic drug delivery system. Colloids Surf., B 2017, 157, 118–129. 10.1016/j.colsurfb.2017.05.061. 28578270

[ref24] Wang F. C.; Miyazaki Y.; Marangoni A. G. Nanostructured oil in cosmetic paraffin waxes. Cryst. Growth Des. 2018, 18, 2677–2680. 10.1021/acs.cgd.8b00042.

[ref25] Xu X. Improve Bioaccessibility of Quercetin Using Pseudo-organogel Based Nanoemulsions. Rutgers University-Graduate School-New Brunswick, 2014.

[ref26] Ghiasi F.; Eskandari M. H.; Golmakani M.-T.; Hosseini S. M. H. Development of highly stable colloidal dispersions of gelled-oil nanoparticles loaded with cuminaldehyde. J. Colloid Interface Sci. 2019, 541, 65–74. 10.1016/j.jcis.2019.01.010. 30682594

[ref27] Field S.; Newton-Bishop J. A. Melanoma and vitamin D. OA Mol. Oncol. 2011, 5, 197–214. 10.1016/j.molonc.2011.01.007. PMC5528277 21371954

[ref28] Luo Y.; Teng Z.; Wang Q. Development of zein nanoparticles coated with carboxymethyl chitosan for encapsulation and controlled release of vitamin D3. J. Agric. Food Chem. 2012, 60, 836–843. 10.1021/jf204194z. 22224939

[ref29] Gueli N.; Verrusio W.; Linguanti A.; Di Maio F.; Martinez A.; Marigliano B.; Cacciafesta M. Vitamin D: drug of the future. A new therapeutic approach. Arch. Gerontol. Geriatr. 2012, 54, 222–227. 10.1016/j.archger.2011.03.001. 21458871

[ref30] Ballard J. M.; Zhu L.; Nelson E. D.; Seburg R. A. Degradation of vitamin D3 in a stressed formulation: the identification of esters of vitamin D3 formed by a transesterification with triglycerides. J. Pharmaceut. Biomed. Anal. 2007, 43, 142–150. 10.1016/j.jpba.2006.06.036. 16901672

[ref31] Meghani N.; Patel P.; Kansara K.; Ranjan S.; Dasgupta N.; Ramalingam C.; Kumar A. Formulation of vitamin D encapsulated cinnamon oil nanoemulsion: Its potential anti-cancerous activity in human alveolar carcinoma cells. Colloids Surf., B 2018, 166, 349–357. 10.1016/j.colsurfb.2018.03.041. 29631227

[ref32] Mahdi Jafari S.; Masoudi S.; Bahrami A. A Taguchi approach production of spray-dried whey powder enriched with nanoencapsulated vitamin D3. Dry. Technol. 2019, 37, 2059–2071. 10.1080/07373937.2018.1552598.

[ref33] Bochicchio S.; Barba A. A.; Grassi G.; Lamberti G. Vitamin delivery: Carriers based on nanoliposomes produced via ultrasonic irradiation. LWT - Food Sci. Technol. 2016, 69, 9–16. 10.1016/j.lwt.2016.01.025.

[ref34] Mohammadi M.; Pezeshki A.; Mesgari Abbasi M.; Ghanbarzadeh B.; Hamishehkar H. Vitamin D3-loaded nanostructured lipid carriers as a potential approach for fortifying food beverages; in vitro and in vivo evaluation. Adv. Pharmaceut. Bull. 2017, 7, 61 10.15171/apb.2017.008. PMC5426735 28507938

[ref35] Patel M. R.; San Martin-Gonzalez M. F. Characterization of ergocalciferol loaded solid lipid nanoparticles. J. Food Sci. 2012, 77, N8–N13. 10.1111/j.1750-3841.2011.02517.x. 22260120

[ref36] Gahruie H. H.; Niakousari M.; Parastouei K.; Mokhtarian M.; Eş I.; Mousavi Khaneghah A. Co-encapsulation of vitamin D3 and saffron petals’ bioactive compounds in nanoemulsions: Effects of emulsifier and homogenizer types. J. Food Process. Preserv. 2020, 44, e14629 10.1111/jfpp.14629.

[ref37] Glowka E.; Stasiak J.; Lulek J. Drug delivery systems for vitamin D supplementation and therapy. Pharmaceutics 2019, 11, 347 10.3390/pharmaceutics11070347. PMC6680748 31323777

[ref38] Lopes N. A.; Barreto Pinilla C. M.; Brandelli A. Antimicrobial activity of lysozyme-nisin co-encapsulated in liposomes coated with polysaccharides. Food Hydrocolloids 2019, 93, 1–9. 10.1016/j.foodhyd.2019.02.009.

[ref39] Giacomozzi A. S.; Palla C. A.; Carrín M. E.; Martini S. Physical Properties of Monoglycerides Oleogels Modified by Concentration, Cooling Rate, and High-Intensity Ultrasound. J. Food Sci. 2019, 84, 2549–2561. 10.1111/1750-3841.14762. 31433063

[ref40] Lopez-Polo J.; Silva-Weiss A.; Giménez B.; Cantero-López P.; Vega R.; Osorio F. A. Effect of lyophilization on the physicochemical and rheological properties of food grade liposomes that encapsulate rutin. Food Res. Int. 2020, 130, 108967 10.1016/j.foodres.2019.108967. 32156401

[ref41] Vélez M. A.; Perotti M. C.; Zanel P.; Hynes E. R.; Gennaro A. M. Soy PC liposomes as CLA carriers for food applications: Preparation and physicochemical characterization. J. Food Eng. 2017, 212, 174–180. 10.1016/j.jfoodeng.2017.06.001.

[ref42] Umagiliyage A. L.; Becerra-Mora N.; Kohli P.; Fisher D. J.; Choudhary R. Antimicrobial efficacy of liposomes containing d-limonene and its effect on the storage life of blueberries. Postharvest Biol. Technol. 2017, 128, 130–137. 10.1016/j.postharvbio.2017.02.007.

[ref43] Cruz A.; Marsh D.; Pérez-Gil J. Rotational dynamics of spin-labelled surfactant-associated proteins SP-B and SP-C in dipalmitoylphosphatidylcholine and dipalmitoylphosphatidylglycerol bilayers. Biochim. Biophys. Acta 1998, 1415, 125–134. 10.1016/s0005-2736(98)00182-5. 9858708

[ref44] Rocha J. C. B.; Lopes J. D.; Mascarenhas M. C. N.; Arellano D. B.; Guerreiro L. M. R.; da Cunha R. L. Thermal and rheological properties of organogels formed by sugarcane or candelilla wax in soybean oil. Food Res. Int. 2013, 50, 318–323. 10.1016/j.foodres.2012.10.043.

[ref45] Gahruie H. H.; Eskandari M. H.; Khalesi M.; Van der Meeren P.; Hosseini S. M. H. Rheological and interfacial properties of basil seed gum modified with octenyl succinic anhydride. Food Hydrocolloids 2020, 101, 105489 10.1016/j.foodhyd.2019.105489.

[ref46] Sila M.; Au S.; Weiner N. Effects of Triton X-100 concentration and incubation temperature on carboxyfluorescein release from multilamellar liposomes. Biochim. Biophys. Acta 1986, 859, 165–170. 10.1016/0005-2736(86)90211-7.

[ref47] Kragh-Hansen U.; le Maire M.; Møller J. V. The mechanism of detergent solubilization of liposomes and protein-containing membranes. Biophys. J. 1998, 75, 2932–2946. 10.1016/s0006-3495(98)77735-5. 9826614 PMC1299965

[ref48] Yang G.; Yang T.; Zhang W.; Lu M.; Ma X.; Xiang G. In vitro and in vivo antitumor effects of folate-targeted ursolic acid stealth liposome. J. Agric. Food Chem. 2014, 62, 2207–2215. 10.1021/jf405675g. 24528163

[ref49] Kakami Y.; Takeuchi I.; Makino K. Percutaneous immunization with 40-nm antigen-encapsulated elastic liposomes. Colloids Surf., A 2019, 566, 128–133. 10.1016/j.colsurfa.2019.01.023.

[ref50] Yusuf A.; Casey A. Evaluation of silver nanoparticle encapsulation in DPPC-based liposome by different methods for enhanced cytotoxicity. Int. J. Polym. Mater. Polym. Biomater. 2019, 69, 860–871. 10.1080/00914037.2019.1626390.

[ref51] Sabín J.; Prieto G.; Ruso J. M.; Hidalgo-Alvarez R.; Sarmiento F. Size and stability of liposomes: a possible role of hydration and osmotic forces. Eur. Phys. J. E: Soft Matter Biol. Phys. 2006, 20, 401–408. 10.1140/epje/i2006-10029-9. 16957831

[ref52] Liu W.; Liu W.; Ye A.; Peng S.; Wei F.; Liu C.; Han J. Environmental stress stability of microencapsules based on liposomes decorated with chitosan and sodium alginate. Food Chem. 2016, 196, 396–404. 10.1016/j.foodchem.2015.09.050. 26593507

[ref53] Cheng C.; Wu Z.; McClements D. J.; Zou L.; Peng S.; Zhou W.; Liu W. Improvement on stability, loading capacity and sustained release of rhamnolipids modified curcumin liposomes. Colloids Surf., B 2019, 183, 110460 10.1016/j.colsurfb.2019.110460. 31473408

[ref54] Cheng C.; Peng S.; Li Z.; Zou L.; Liu W.; Liu C. Improved bioavailability of curcumin in liposomes prepared using a pH-driven, organic solvent-free, easily scalable process. RSC Adv. 2017, 7, 25978–25986. 10.1039/c7ra02861j.

[ref55] Kontogiannopoulos K. N.; Dasargyri A.; Ottaviani M. F.; Cangiotti M.; Fessas D.; Papageorgiou V. P.; Assimopoulou A. N. Advanced drug delivery nanosystems for Shikonin: a calorimetric and electron paramagnetic resonance study. Langmuir 2018, 34, 9424–9434. 10.1021/acs.langmuir.8b00751. 30032619

[ref56] Martini G.; Ciani L. Electron spin resonance spectroscopy in drug delivery. Phys. Chem. Chem. Phys. 2009, 11, 211–254. 10.1039/b808263d. 19088979

[ref57] Chen C.; Tang H.-R.; Sutcliffe L. H.; Belton P. S. Green tea polyphenols react with 1, 1-diphenyl-2-picrylhydrazyl free radicals in the bilayer of liposomes: direct evidence from electron spin resonance studies. J. Agric. Food Chem. 2000, 48, 5710–5714. 10.1021/jf000807a. 11087543

[ref58] Pincelli M. M.; Levstein P. R.; Fidelio G. D.; Gennaro A. M. Cholesterol-induced alterations of the packing properties of gangliosides: an EPR study. Chem. Phys. Lipids 2000, 104, 193–206. 10.1016/s0009-3084(99)00127-9. 10669311

[ref59] Coderch L.; Fonollosa J.; De Pera M.; Estelrich J.; De La Maza A.; Parra J. L. Influence of cholesterol on liposome fluidity by EPR: relationship with percutaneous absorption. J. Controlled Release 2000, 68, 85–95. 10.1016/s0168-3659(00)00240-6. 10884582

[ref60] Zhang T.; Su M.; Jiang X.; Xue Y.; Zhang J.; Zeng X.; Wu Z.; Guo Y.; Pan D. Transepithelial transport route and liposome encapsulation of milk-derived ACE-inhibitory peptide Arg-Leu-Ser-Phe-Asn-Pro. J. Agric. Food Chem. 2019, 67, 5544–5551. 10.1021/acs.jafc.9b00397. 31007021

[ref61] Tai K.; Rappolt M.; He X.; Wei Y.; Zhu S.; Zhang J.; Mao L.; Gao Y.; Yuan F. Effect of β-sitosterol on the curcumin-loaded liposomes: Vesicle characteristics, physicochemical stability, in vitro release and bioavailability. Food Chem. 2019, 293, 92–102. 10.1016/j.foodchem.2019.04.077. 31151654

[ref62] Mohammadi M.; Ghanbarzadeh B.; Hamishehkar H. Formulation of nanoliposomal vitamin D3 for potential application in beverage fortification. Adv. Pharmaceut. Bull. 2014, 4, 569–575. 10.5681/apb.2014.084. PMC4312407 25671191

[ref63] Hanabusa K.; Suzuki M. Development of low-molecular-weight gelators and polymer-based gelators. Polym. J. 2014, 46, 776–782. 10.1038/pj.2014.64.

